# Combination of High Ankle–Brachial Index and Hard Coronary Heart Disease Framingham Risk Score in Predicting the Risk of Ischemic Stroke in General Population

**DOI:** 10.1371/journal.pone.0106251

**Published:** 2014-09-08

**Authors:** Yinyin Zhang, Jie Chen, Kun Zhang, Tong Wang, Minyi Kong, Renhua Chen, Yu Liu, Jianping Chen, Zhiyu Wang, Jingfeng Wang, Hui Huang

**Affiliations:** 1 Department of Cardiology, Sun Yat-sen Memorial Hospital of Sun Yat-sen University, Guangzhou, Guangdong Province, China; 2 Guangdong Province Key Laboratory of Arrhythmia and Electrophysiology, Guangzhou, Guangdong Province, China; 3 Department of Radiation Oncology, Sun Yat-sen Memorial Hospital of Sun Yat-sen University, Guangzhou, Guangdong Province, China; 4 School of Chinese Medicine, The University of Hong Kong, Hong Kong, China; University of Virginia, United States of America

## Abstract

Our previous study showed that the patients with more metabolic risk factors had higher risk of high ankle–brachial index (ABI), but the relationship between high ABI and the risk of severe cardiovascular and cerebrovascular diseases is still under debate. This study aims to evaluate this association in the general population. 1486 subjects of South China were recruited in the study. 61 subjects were defined as high ABI group (ABI≥1.3) and 65 subjects were randomly selected as normal ABI group (0.9<ABI<1.3). Biochemical parameters, clinical characteristics and 10-year hard coronary heart disease (HCHD) Framingham Risk Score (FRS) were compared between two groups. The results showed that the 10-year HCHD FRS of high ABI group was significantly higher than normal ABI group (7.87±6.11 vs. 3.98±2.90%, *P*<0.001). There was a positive correlation between ABI value and HCHD FRS in overweight participants (R = 0.576, P<0.01). The prevalence of ischemic stroke was higher in high ABI group than normal ABI group (21.3% vs. 6.2%, *P*<0.05), and it was higher in participants with HCHD FRS≥6% than those with HCHD FRS<6% (19.1% vs. 6.9%, P<0.05). Moreover, the prevalence of ischemic stroke was higher in participants with high ABI and HCHD FRS≥6% than those with normal ABI and HCHD FRS<6% (26.7% vs. 4.1%, *P<0.05*). BMI, hypertension, hsCRP and smoking were proved to be the independent factors and effective predictors for high ABI (*P<0.05*). In conclusion, high ABI combined with high HCHD FRS should be a potential predictor of ischemic stroke in the general population of South China.

## Introduction

Ankle-brachial index (ABI) is a simple and noninvasive method which is widely used to identify peripheral artery disease. High ABI (≥1.3) indicates stiff distal arteries, probably in relation with the medial arterial calcification (MAC) [1], [2]. Our previous study showed that patients with more metabolic risk factors had higher risk of high ABI [3], but it is controversial that whether high ABI will increase the risk of cardiovascular and cerebrovascular events in the general population. The Strong Heart Study(SHS) was the first large prospective study to show that the American Indians with high ABI had increased all-cause and cardiovascular disease (CVD) mortality [4]. Other study evidence also demonstrated that high ABI was independently associated with incident CVD events defined as coronary disease, stroke, or other atherosclerotic CVD death [5], [6]. However, the Atherosclerosis Risk in Communities (ARIC) Study did not support this conclusion and showed that white and black men and women with high ABI did not suffer greater CVD event rates than those with normal ABI [7]. A recent study found that coronary artery calcium (CAC) volume was positively and independently associated with CVD risk. But CAC density was inversely and significantly associated with CVD risk [8]. For high ABI usually indicated the existence of arterial calcification, the relationship between high ABI and the risk of cardiovascular and cerebrovascular events in the general population needs to be further explored.

Ischemic stroke incidence is significantly higher among Chinese than white populations and the elevated incidence of stroke is a great challenge for the public health [9]. Bouchi et al. found that the type 2 diabetic patients with high ABI had increased risk of silent cerebral infarction independently of other traditional cardiovascular risk factors [10]. Albert et al. demonstrated that intracranial arterial calcification was highly prevalent in hemodialysis patients, especially in those with acute ischemic stroke [11]. But the association between high ABI and ischemic stroke in the general population has not been examined to date.

The sudden onset of hard coronary heart disease (HCHD) is hard to be predicted. The 10-year HCHD Framingham Risk Score(FRS) is a received standard to predict the risk of myocardial infarction and coronary death in individuals free of coronary heart disease(CHD) and diabetes mellitus(DM). We evaluated the predictive value of cardiovascular and cerebrovascular events by the 10-year HCHD FRS in the general population of South China with high ABI. Meanwhile, studies on the independent factors of high ABI in the general population are extremely limited. And the effective predictors for high ABI remain unclear.

Because high ABI has a close correlation with ischemic stroke and HCHD FRS is an effective standard to predict severe cardiovascular events, we suppose that combination of elevated ABI and HCHD FRS is important to predict ischemic stroke in general population. And we validated this hypothesis in this study. We simultaneously compared the related clinical index to determine the independent risk factors and predictors for high ABI.

## Methods

### Ethics statement

The study protocol conformed to the ethical guidelines of the 1975 Declaration of Helsinki as reflected in a priori approval by the Ethics Committee of Sun Yat-sen Memorial Hospital of Sun Yat-sen University. Written Informed consent was obtained from each participant.

### Study population

This study is a cross-sectional study. 1604 people in South China who came to the Sun Yat-sen Memorial Hospital for either routine physical examinations or hospitalizations were enrolled in this study between November 2009 and August 2012. All participants completed a detailed health history questionnaire. Individuals with CHD, DM, overt congestive heart failure, peripheral artery disease, familial hyperlipidemia, severe hepatic dysfunction and renal insufficiency, potential infectious, inflammatory diseases, immunologic diseases, carcinoma, corticosteroid therapy, or with an age of over 80 years or below 20 years were excluded from this study. 118 participants were excluded from this study while 1486 participants who met the inclusion criteria were enrolled in the study. All the participants remained as anonymous.

All of the 1486 participants who enrolled in this study underwent ABI test. Among these participants, 61 members had ABI≥1.3 and were defined as high ABI group. 75 members had ABI<0.9 and the other 1350 members had normal ABI (0.9<ABI<1.3). Because ABI<0.9 is an index to diagnose the peripheral artery disease, the participants with ABI<0.9 were excluded from this study. The prevalence of high ABI was 4.10% in this study population. 65 age-matched and gender-matched participants were randomly selected from all the participants with normal ABI value as normal ABI group using a central selection method generated by a computer program [12].

### Collection of clinical and laboratory parameters

All participants underwent an interview for collection of information regarding the diagnosis and treatment of related diseases, including DM, CHD, hypertension and ischemic stroke, lifestyle factors including smoking habits, and physical examination including assessment of arterial blood pressure, BMI and ABI. The 10-year risks of severe CVD events of participants in two groups were evaluated using the 10-year HCHD FRS [13].

Laboratory parameters including uric acid(UA), alkaline phosphatase (ALP), creatinine(Cr), calcium(Ca), phosphate (P), high-sensitivity C-reactive protein (hsCRP), total cholesterol(TC), triglycerides (TG), high-density lipoprotein cholesterol (HDL-C), low-density lipoprotein cholesterol (LDL-C), apolipoprotein A (apoA), apolipoprotein B(apoB), fasting plasma glucose(FPG) and cholinesterase (CHE) were measured using blood samples drawn by venipuncture after at least 10 hours of overnight fasting. Venous serum samples were measured by a standardized and certified program using automatic biochemical analyzer (7170A, HITACHI, Japan).

### Measurement of ABI

For measurement of ABI, room temperature was constantly kept at 25°C, and one well-trained examiner performed ABI measurements for all participants after 10 minutes' bed rest. The ABI was measured with a non-invasive vascular screening device (VP-1000, OMRON, Japan) using the recommended process by the manufacturer. The systolic pressure (SBP) was measured in the posterior tibial and dorsal pedal arteries of both legs and in the brachial artery of both arms. The ABI was calculated for each leg by dividing the highest lower limb SBP value by the highest upper limb SBP value. Normal ABI was defined by normal values (0.9–1.3). Participants who had an ABI value≥1.3 in either leg were defined as having high ABI [14]. Participants who had one leg with low ABI whereas the other leg with high ABI, were excluded from this study.

### Diagnosis of comorbidities

Hypertension was defined as SBP≥140 mmHg and/or diastolic blood pressure (DBP)≥90 mmHg on at least 3 different occasions, or by a previous diagnosis of hypertension with current antihypertensive medication [15]. Body mass index (BMI) was calculated from values as body weight (kg) divided by the square of the height (m^2^) and overweight was defined as a BMI≥24.0 kg/m^2^ [16]. Ischemic stroke was defined as an acute neurological event lasting more than 24 hours associated with evidence of ischemic focus of brain in computer tomography or magnetic resonance imaging.

### Statistical analysis

According to normality test results, data were presented as frequencies for categorical variables, mean values with SD for normally distributed continuous variables and median values with 25% and 75% percentiles for ordinal variables. The following data were compared between two groups with methods of the independent samples t-test for normally distributed data, Mann–Whitney U test for non-normally distributed data and Pearson chi-square for categorical variables. Correlation analysis was used to analyze the correlation between ABI value and 10-year HCHD FRS in normal weight and overweight participants. Independent factors of high ABI were identified by binary logistic regression with the enter method. Receiver Operating Characteristics (ROC) curve analysis was used to quantify the predictive values of independent parameters for high ABI. For all the statistical tests, two-tailed P-value <0.05 implied the rejection of the null hypothesis and the results were considered statistically significant. All statistical analyses were performed using the software SPSS 16.0.

## Results

### Demographic and clinical characteristics of normal and high ABI groups

Demographic and biochemical data of normal and high ABI groups are shown in Table 1. Compared with normal ABI group, participants in high ABI group had larger BMI values (25.88±3.18 vs. 24.50±2.25, P<0.05). The level of hsCRP was 2.92(1.38–8.40) for high ABI group and 1.78(0.91–3.18) for normal ABI group. The level of hsCRP was significantly higher in high ABI group than normal ABI group (P<0.01). 65.6% of participants in the high ABI group had a history of smoking, while only 35.4% of participants in the normal ABI group had a history of smoking. There was a significant difference in smoking history between these two groups (P<0.05). And the prevalence of hypertension in high ABI group also markedly elevated (55.7% vs. 36.9%, P<0.05). Interestingly, the differences of TC, TG, LDL-C and HDL-C were not significantly different between these two groups (P>0.05).

**Table 1:** Comparison of the demographic and biochemical data between the high and normal ABI groups.

Variable	High ABI group	Normal ABI group	P
Number(n)	61	65	-
Age(year)	58±12	58±10	0.90
Male gender(%)	49.2	46.2	0.73
Smoking(%)	65.6	35.4	0.001*
BMI(kg/m2)	25.88±3.18	24.50±2.25	0.01*
ALP (U/L)	69.79±21.89	70.86±30.45	0.82
Ca(mmol/L)	2.26±0.14	2.29±0.13	0.19
P(mmol/L)	1.19±0.20	1.18±0.19	0.84
TC(mmol/L)	5.08±1.24	5.14±0.98	0.77
LDL-C(mmol/L)	3.03±0.87	3.12±0.84	0.55
HDL-C(mmol/L)	1.21±0.31	1.25±0.28	0.46
TG(mmol/L)	2.08(1.42–2.89)	1.84(1.62–2.33)	0.67
Apo A(g/L)	1.18±0.24	1.19±0.22	0.81
HsCRP(mg/L)	2.92(1.38–8.40)	1.78(0.91–3.18)	0.07
Cr(µmol/L)	99.98±21.71	102.06±18.77	0.57
UA(µmol/L)	398.16±104.95	419.82±99.11	0.88
FPG(mmol/L)	5.30(4.70–5.70)	5.30(5.00–5.90)	0.46
CHE(U/L)	9084.70±1836.28	9606.90±2043.66	0.14
Hypertension(%)	55.7	36.9	0.03*

Values are expressed as mean ± S.D., median (25%–75% percentiles) or as percentage (%) of patients. P-value based on Pearson chi-square for categorical variables and t-test for normally distributed data is for comparison between the high and normal ABI groups (*P<0.05). Mann–Whitney U test is used to compare nonnormally distributed variables between the high and normal ABI groups (*P<0.01). Abbreviations: ABI, ankle-brachial index; BMI, body mass index; ALP, alkaline phosphatase; Ca, calcium; P, phosphate; Cr, creatinine; UA, uric acid; TC, total cholesterol; LDL-C, low-density lipoprotein cholesterol; HDL-C, high-density lipoprotein cholesterol; TG, triglycerides; apo, apolipoprotein; hsCRP, high-sensitivity C-reactive protein; FPG, fasting plasma glucose; CHE, cholinesterase.

### The 10-year risks of HCHD of normal and high ABI groups

**Figure 1:**
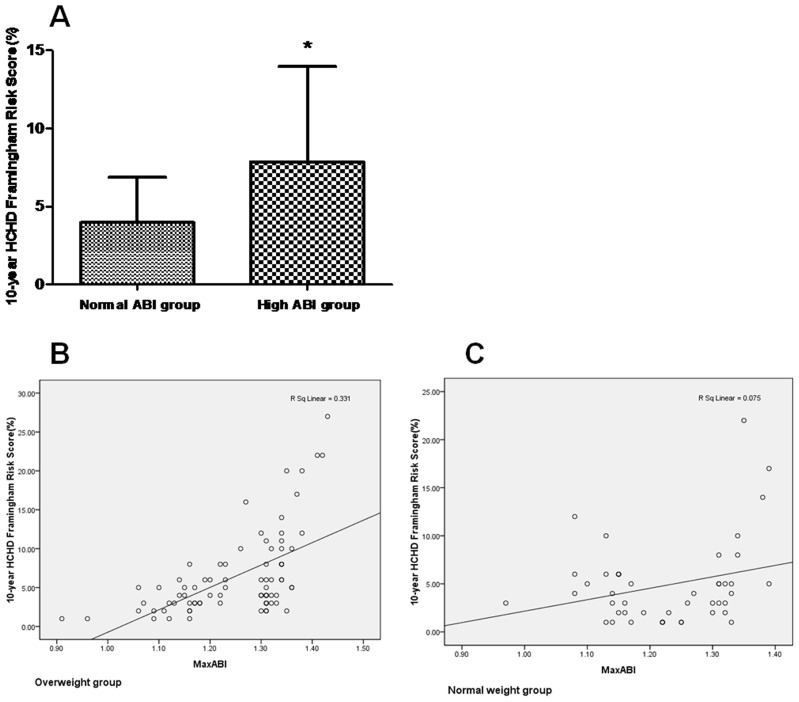
Comparison of the 10-year HCHD FRS between the normal and high ABI groups (*P<0.001 vs. normal ABI group) (A), correlation between ABI value and the 10-year HCHD FRS in overweight participants (B) and correlation between ABI value and the 10-year HCHD FRS in normal weight participants (C).

As shown in Figure 1A, the 10-year HCHD FRS of high ABI group was 7.87±6.11% and that of normal ABI group was 3.98±2.90%. There was a significant difference in the 10-year HCHD FRS between these two groups (P<0.001).

### Correlation between ABI value and 10-year HCHD FRS in overweight and normal weight participants

All the participants in the normal and high ABI groups were divided into normal weight and overweight groups according to the BMI value. And there was a positive correlation between ABI value and 10-year HCHD FRS in the overweight participants (R = 0.576, P<0.01, Figure 1B). Nevertheless, there was no significant correlation between ABI value and 10-year HCHD FRS in the normal weight participants (R = 0.273, P>0.05, Figure 1C).

### The comparison of prevalence of ischemic stroke according to the ABI value and 10-year HCHD FRS

**Figure 2:**
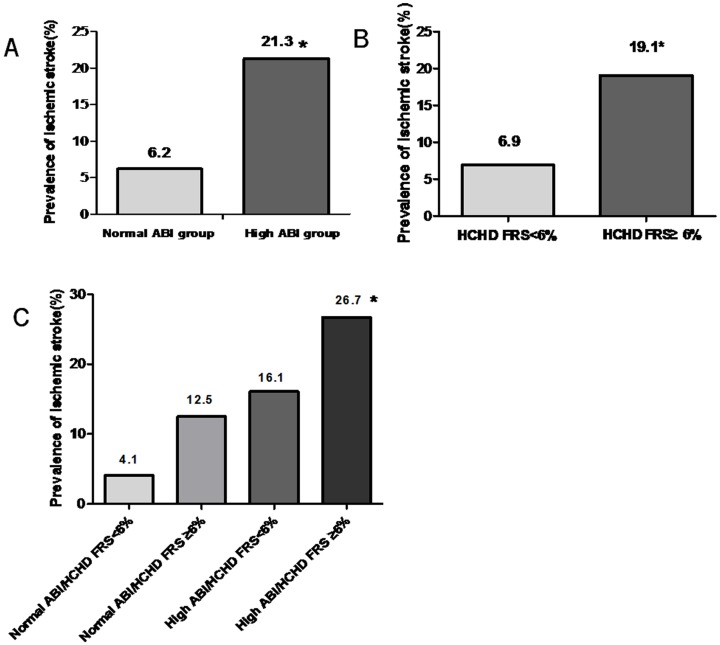
Comparison of the prevalence of ischemic stroke between the normal and high ABI groups (*P<0.05 vs. normal ABI group) (A), comparison of the prevalence of ischemic stroke between the group with HCHD FRS≥6% and that with HCHD FRS<6% (*P<0.05 vs. HCHD FRS<6% group) (B) and the prevalence of ischemic stroke in participants with different ABI value and 10-year HCHD FRS (*P<0.05 vs. normal ABI/HCHD FRS<6% group) (C).

The prevalence of ischemic stroke was significantly higher in high ABI group than normal ABI group (21.3% vs. 6.2%, P<0.05, Figure 2A). Because the mean value of HCHD FRS of all the subjects in this study was close to 6% and HCHD FRS<6% was regarded as a low level of FRS [17], we selected HCHD FRS = 6% as the cut-off point when grouping. And all the subjects of this study were also divided into two groups according to 10-year HCHD FRS. The prevalence of ischemic stroke was compared between these two groups and it was proved that the prevalence of ischemic stroke in the group with HCHD FRS≥6% was higher than the group with HCHD FRS<6%(19.1% vs. 6.9%, P<0.05, Figure 2B). Furthermore, the participants were divided into four groups according to the ABI value and 10-year HCHD FRS. The prevalence of ischemic stroke was compared between these four groups and the results showed that the prevalence of ischemic stroke in the group with high ABI and HCHD FRS≥6% was higher than the group with normal ABI and HCHD FRS<6% (26.7% vs. 4.1%, P<0.05, Figure **2C**), which suggested that high ABI combined with high HCHD FRS indicated elevated risk of ischemic stroke in the general population. And it was shown that the prevalence of ischemic stroke in female participants with high ABI and HCHD FRS≥6% was 27.8% and that of the male participants with high ABI and HCHD FRS≥6% was 25.0%. The difference of ischemic stroke rates between these two groups was not significant (P>0.05).

### Regression analysis of the independent risk factors for high ABI

Binary logistic regression analysis with the enter method was used to identify the independent risk factors for high ABI (Table 2). BMI (OR 1.206; 95% CI 1.050–1.384; P<0.05), hsCRP (OR 1.246; 95% CI 1.088–1.427; P<0.05), smoking (OR 0.288; 95% CI 0.138–0.598; P<0.01) and hypertension (OR 0.435; 95% CI 0.213–0.889; P<0.05) were all proved to be the independent risk factors for high ABI. And gender, age, blood lipid could not be identified as the independent factors for high ABI (P>0.05).

**Table 2:** The binary logistic regression analysis of independent risk factors of high ABI by an unadjusted model.

Variable	B	OR	95%CI	P
Age	−0.002	0.998	0.967–1.030	0.90
Male gender	−0.121	0.886	0.440–1.783	0.73
Smoking	−1.247	0.288	0.138–0.598	0.00*
BMI	0.187	1.206	1.050–1.384	0.01*
ALP	−0.002	0.998	0.985–1.012	0.82
Ca	−1.767	0.171	0.012–2.461	0.19
P	0.190	1.209	0.201–7.289	0.84
TC	−0.048	0.953	0.694–1.309	0.77
LDL-C	−0.126	0.882	0.583–1.333	0.55
HDL-C	−0.457	0.633	0.190–2.110	0.46
TG	0.272	1.313	0.850–2.028	0.22
Apo A	−0.187	0.830	0.180–3.827	0.07
HsCRP	0.220	1.246	1.088–1.427	0.00*
Cr	−0.005	0.995	0.978–1.012	0.56
UA	−0.002	0.998	0.994–1.001	0.24
FPG	−0.041	0.960	0.703–1.312	0.80
CHE	0.000	1.000	1.000–1.000	0.14
Hypertension	−0.883	0.435	0.213–0.889	0.02*

Binary logistic regression analysis with the enter method is used to assess the independent risk factors of high ABI in unadjusted model (*P<0.05). Abbreviations: ABI, ankle-brachial index; BMI, body mass index; ALP, alkaline phosphatase; Ca, calcium; P, phosphate; Cr, creatinine; UA, uric acid; TC, total cholesterol; LDL-C, low-density lipoprotein cholesterol; HDL-C, high-density lipoprotein cholesterol; TG, triglycerides; apo, apolipoprotein; hsCRP, high-sensitivity C-reactive protein; FPG, fasting plasma glucose; CHE, cholinesterase.

### Predictor analysis for high ABI with receiver operating characteristic curve

**Figure 3:**
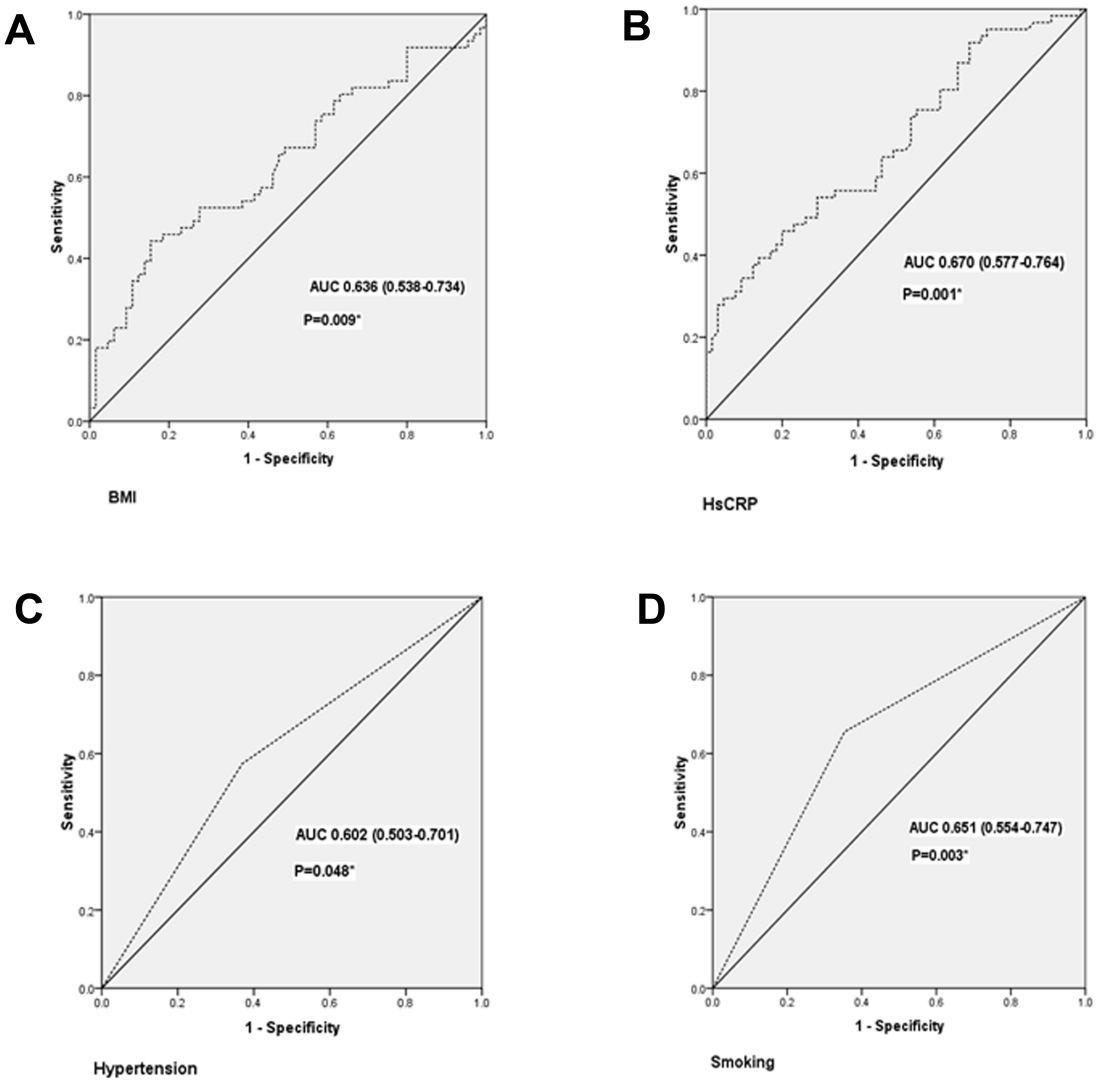
Receiver-operating characteristic (ROC) curve for factors in predicting high ABI (*P<0.05). BMI, body mass index; hsCRP, high-sensitivity C-reactive protein; AUC, area under the curve.

The ROC analysis was used to analyze whether the above risk factors was the predictors for high ABI (Figure 3). The area under the curve (AUC) was 0.636 (95% CI  = 0.538–0.734) for BMI (Figure 3A), 0.670 (95% CI  = 0.577–0.764) for hsCRP (Figure 3B), 0.602 (95% CI  = 0.503–0.701) for hypertension (Figure 3C) and 0.651 (95% CI  = 0.554–0.747) for smoking (Figure 3D). Smoking, hypertension, high level of BMI and hsCRP were all shown to be the appropriate predictors for high ABI (P<0.05). HsCRP, with a threshold value of 0.92, provided 95.1% sensitivity and 26.2% specificity. And BMI, with a threshold value of 23.53, provided 80.3% sensitivity and 36.9% specificity for the prediction of high ABI.

## Discussion

Our study found that the general population with high ABI had elevated risk of severe cardiovascular events and ischemic stroke. Furthermore, it was demonstrated that high ABI combined with high HCHD FRS was helpful to predict the risk of ischemic stroke.

Our study demonstrated that the prevalence of high ABI in the general population of South China was 4.10%. But Helaine et al. showed that the prevalence of high ABI in 4393 American Indians aged 45–74 was 9.2% [4]. The participants in our study were the general population of South China aged 20–80. In the study of Helaine et al., the advanced age and different race of the participants might result in higher prevalence of high ABI than our study. The relationship between high ABI and the risk of CVD events is under debate [4]–[7]. The different results of the above studies might be due to different significance of ABI in different races. Moreover, data from the general population are not available. Thus, it is controversial that whether high ABI will increase the risk of cardiovascular events in the general population and the relationship between high ABI and CVD risk needs to be further studied. The 10-year HCHD FRS is a commonly used standard to predict the risk of severe cardiovascular events. Our study is the first one to use the 10-year HCHD FRS to evaluate the risk of HCHD in the general population with high ABI. The results showed that 10-year HCHD FRS in South China participants with high ABI was nearly two times higher than those in normal ABI group. Furthermore, the ABI value had a positive correlation with HCHD FRS in the overweight participants. These findings further indicated that people in South China with high ABI had increased 10-year risk of myocardial infarction or death from CHD. And the overweight participants with larger ABI value were susceptible to HCHD. Some studies proved that high ABI was independently associated with increased CVD risk [4]–[6]. Otherwise, the Atherosclerosis Risk in Communities (ARIC) Study found that white and black men and women with high ABI did not have higher CVD event rates than those with normal ABI [7]. Our study supported the conclusion that high ABI had a close correlation with CVD risk in the general population of South China and might be helpful to predict the risk of CVD events. The different races of the above studies may result in the different conclusion and larger studies of different races are needed to further demonstrate the value of high ABI in predicting the CVD risk.

Furthermore, our study demonstrated that the proportion of ischemic stroke in high ABI group was nearly four times higher than those in normal ABI group. Allison et al. also found that high ABI was strongly associated with ischemic stroke in patients with the age >50 years [18]. Our study further demonstrated that high ABI increased the risk of ischemic stroke in the general population. 10-year HCHD FRS is a received standard to predict severe cardiovascular events, and we found that the participants with high ABI had higher HCHD FRS than those with normal ABI. It was also demonstrated that participants with HCHD FRS≥6% had higher prevalence of ischemic stroke than those with HCHD FRS<6%, which indicated that 10-year HCHD FRS was a useful standard to predict cerebrovascular disease. In order to identify whether combination of ABI and 10-year HCHD FRS was helpful to discern the participants with elevated risk of ischemic stroke, we divided the subjects into four groups according to the ABI value and HCHD FRS. And the results showed that the prevalence of ischemic stroke in the group with high ABI and HCHD FRS≥6% was significantly higher than the group with normal ABI and HCHD FRS<6%. Moreover, there was no significant difference in the prevalence of ischemic stroke between male and female participants with high ABI and HCHD FRS≥6%. And it was indicated that combination of ABI and HCHD FRS can predict ischemic stroke equally well between male and female participants. These findings suggested that higher HCHD FRS indicated higher risk of ischemic stroke in participants with high ABI. The combination of ABI value and HCHD FRS was an independent predictor for ischemic stroke in the general population of South China.

In this study, we investigated whether the traditional risk factors of atherosclerosis were also involved in the progression of high ABI. Hypertension and smoking were proved to be the risk factors of high ABI. These findings were similar to several studies [7], [12], [18], [19]. Moreover, it was verified that BMI markedly elevated in high ABI group. Several studies also found that patients with high ABI had elevated BMI [12], [20]. But a recent study found that patients with high ABI did not have larger BMI than those with normal ABI, which only enrolled patients if they were 70 years or older or if they were age 50 to 69 years and had a history of at least 10-years of smoking or diabetes or both [18]. However, age, male gender and hyperlipidemia were not shown to be the risk factors of high ABI in our study. Participants of different races aged 45 to 84 years old were recruited in the study of Allison et al. [20]. They found that hyperlipidemia was not associated with high ABI, while male gender was associated with high ABI. But this study enrolled subjects of different race, our study subjects were the general population of South China. Different study populations might result in the diversities of the risk factors.

Furthermore, our study demonstrated that the increased inflammation marker of hsCRP was associated with high ABI. Previous studies also showed that inflammatory cytokines significantly increased in patients with high ABI [5], [21]. Recently, there has been increasing appreciation of obesity as a state of chronic inflammation [22]. And CRP was noted as a marker of obesity related inflammation [23]. Considering the increased levels of BMI and hsCRP in participants with high ABI, it is supposed that the obesity-related inflammatory state might have a key role in the progression of high ABI. Further researches are needed to identify the mechanism of this process.

Prediction of high ABI is useful to identify the patients who may benefit from more active treatment to risk factors of high ABI. And it helps to discern the patients with higher risk of severe cardiovascular diseases, and gives better management to them. Our study showed that smoking, BMI, hypertension and hsCRP were effective predictors for high ABI in the people of South China.

Some limitations of our study should be acknowledged. First of all, 10-year HCHD FRS is only a standard to predict the risk of HCHD. So, larger and longer-term follow up studies will be needed to further determine the association between high ABI and the risk of HCHD. In addition, the cross-sectional nature of this study prevents making any formal statements regarding whether certain subclinical disease measured preceded others. Finally, though 1604 subjects participated in this study, a small number of participants were enrolled in the final study for the low incidence of high ABI in the general population of South China. So, larger studies of different races are needed in the future.

## Conclusions

Our study indicated that the general population in South China with high ABI had elevated risk to suffer from major cardiovascular events and was susceptible to ischemic stroke. Moreover, high ABI combined with high HCHD FRS should be a potentially effective index to predict the risk of ischemic stroke and needed to be further studied. Smoking, hypertension, BMI and hsCRP were all the risk factors and promising predictors for high ABI. Hence, the obese people and those with higher level of hsCRP should be reminded of the elevated risk of high ABI. These findings suggested that the participants with high ABI and high HCHD FRS should be alert to increased risk of ischemic stroke.
